# Tumour-to-liver ratio determined by [^68^Ga]Ga-DOTA-TOC PET/CT as a prognostic factor of lanreotide efficacy for patients with well-differentiated gastroenteropancreatic-neuroendocrine tumours

**DOI:** 10.1186/s13550-020-00651-z

**Published:** 2020-06-15

**Authors:** Yong-il Kim, Changhoon Yoo, Seung Jun Oh, Sang Ju Lee, Junho Kang, Hee-Sang Hwang, Seung-Mo Hong, Baek-Yeol Ryoo, Jin-Sook Ryu

**Affiliations:** 1 Department of Nuclear Medicine, Asan Medical Center, University of Ulsan College of Medicine, 88, Olympic-ro 43-gil, Songpa-gu, Seoul, 05505 Republic of Korea; 2 Department of Oncology, Asan Medical Center, University of Ulsan College of Medicine, 88, Olympic-ro 43-gil, Songpa-gu, Seoul, 05505 Republic of Korea; 3 Department of Pathology, Asan Medical Center, University of Ulsan College of Medicine, Seoul, Republic of Korea

**Keywords:** Neuroendocrine tumours, Lanreotide, Somatostatin receptors, Positron emission tomography, Prognosis

## Abstract

**Abstract:**

**Background:**

Lanreotide is a long-acting somatostatin analogue with proven antitumour effects against well-differentiated (WD) gastroenteropancreatic-neuroendocrine tumours (GEP-NETs). However, there are no globally established prognostic factors associated with the efficacy of lanreotide as a treatment for GEP-NETs. We investigated the prognostic value of [^68^Ga]Ga-DOTA-TOC positron emission tomography (PET)/computed tomography (CT) somatostatin receptor imaging for patients with WD GEP-NETs treated with lanreotide.

**Methods:**

In this retrospective study, we included 31 patients with unresectable or metastatic WD GEP-NETs who received lanreotide and underwent [^68^Ga]Ga-DOTA-TOC PET/CT before receiving lanreotide. We captured the following clinicopathological variables: Eastern Cooperative Oncology Group (ECOG) performance status, primary tumour site, NET World Health Organization grade, existence of carcinoid symptoms, previous surgery, previous chemotherapy, and hepatic tumour volume assessed by CT or magnetic resonance imaging (MRI). We also assessed the following [^68^Ga]Ga-DOTA-TOC PET/CT variables: Krenning score, tumour-to-liver ratio (TLR), maximum standardized uptake value (SUVmax), whole tumour volume (WTV), and total receptor expression (TRE, WTV multiplied by SUVmean). The associations between these markers and progression-free survival (PFS) with lanreotide were analysed.

**Results:**

The mean age was 55.1 ± 15.5 years (range 16.0–81.0). The most common primary tumour site was the pancreas, followed by the stomach, and rectum. The median PFS interval with lanreotide was 14.4 months (range 1.3–34.9), with identified disease progression in 20 patients (64.5%). Among the [^68^Ga]Ga-DOTA-TOC PET/CT variables, TLR (< 8.1 vs. ≥ 8.1; *p* = 0.013), SUVmax (< 42.9 vs. ≥ 42.9; *p* = 0.037), and WTV (≥ 58.9 cm^3^ vs. < 58.9 cm^3^; *p* = 0.030) were significantly associated with PFS in the univariate analyses, but only TLR (hazard ratio 3.182 [95% CI 1.189–8.514], *p* = 0.021) remained an independent factor for PFS in the multivariate analysis.

**Conclusions:**

Low TLR, determined via [^68^Ga]Ga-DOTA-TOC PET/CT, can be a factor of worse prognosis in patients with advanced WD GEP-NETs treated with lanreotide.

## Background

Neuroendocrine tumours (NETs) are a heterogeneous group of malignancies arising from neuroendocrine cells throughout the body [1]. Gastroenteropancreatic-NETs (GEP-NETs) are NETs originating from the gastrointestinal tract and pancreas, comprising 60% of all NETs [2]. NETs are rare; however, their incidence has increased more than six-fold in the last 40 years [3]. In the USA, NETs are now the second most common gastrointestinal malignancy type [3]. As most NETs overexpress somatostatin receptors (SSTRs) in their cell membranes, SSTRs are an interesting target for NET therapy and imaging [4].

Examples of SSTR-targeting therapies include somatostatin analogues, such as lanreotide and octreotide [5]. Lanreotide (lanreotide autogel) is a long-acting somatostatin analogue whose antitumour effects against metastatic, well-differentiated (WD) GEP-NETs were proven in the pivotal CLARINET phase III clinical trial [6]. Based on the success of this trial, lanreotide is a globally recommended first-line systemic therapy for metastatic, WD GEP-NETs [7].

The most well-known SSTR-targeting functional imaging techniques use [^68^Ga]Ga-DOTA-conjugated radiotracers ([^68^Ga]Ga-DOTA-TOC, [^68^Ga]Ga-DOTA-TATE, [^68^Ga]Ga-DOTA-NOC, etc.). Among these, [^68^Ga]Ga-DOTA-TOC positron emission tomography (PET)/computed tomography (CT) targets SSTR-2 and SSTR-5 [8]. The roles of [^68^Ga]Ga-DOTA-TOC PET/CT in staging, selecting patients for therapeutic options, and therapy monitoring of the NET patients have been previously investigated [9].

Because [^68^Ga]Ga-DOTA-TOC PET/CT imaging and lanreotide share the same SSTR targets, [^68^Ga]Ga-DOTA-TOC PET/CT may have prognostic implications in association with the efficacy of lanreotide for WD GEP-NETs, and this tool has the advantages of non-invasiveness and wide extent of examination covering whole body, in contrast with pathologic biomarkers. However, the implications of [^68^Ga]Ga-DOTA-TOC PET/CT as a prognostic factor applicable to lanreotide treatment efficacy have not been fully evaluated [10]. We investigated the prognostic value of [^68^Ga]Ga-DOTA-TOC PET/CT for patients with unresectable or metastatic WD GEP-NETs treated with lanreotide.

## Methods

### Patients

**Fig. 1:**
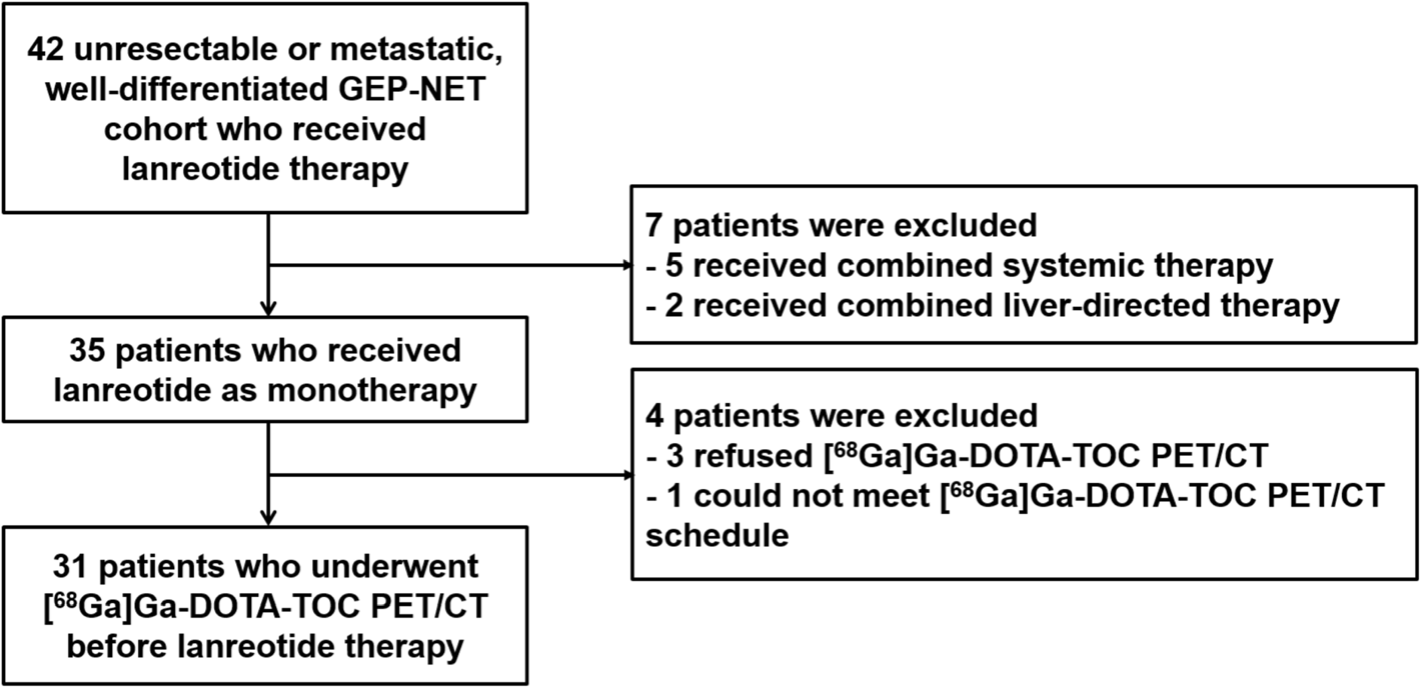
Patient inclusion

We retrospectively evaluated 64 patients with metastatic, well-differentiated GEP-NETs who received lanreotide therapy from September 2016 to May 2018 at Asan Medical Center, Seoul, Republic of Korea. The clinical outcomes of these patients are available elsewhere [11]. Patients who received lanreotide and underwent [^68^Ga]Ga-DOTA-TOC PET/CT before receiving lanreotide were included in this retrospective analysis. Patients who received lanreotide in combination with other systemic agents or local therapy were excluded (Fig. 1). Lanreotide was given to these patients irrespective of [^68^Ga]Ga-DOTA-TOC PET/CT findings. A total of 31 patients who fulfilled the above criteria were included in the present analysis.

The following clinicopathologic variables were assessed via a review of medical records: Eastern Cooperative Oncology Group (ECOG) performance status, primary tumour site, NET World Health Organization (WHO) grade, existence of carcinoid symptoms, previous surgery, previous chemotherapy, and hepatic tumour volume assessed by CT or magnetic resonance imaging (MRI). NETs were graded using the 2017 WHO classification system [12]. Patient profiles and clinical outcomes were obtained from the review of medical records. The study design and waiver of the requirement for informed consent were approved by our institutional review board (IRB) (2019-1484).

### Treatment and response assessment

1Lanreotide was administered subcutaneously every 4 weeks at a dose of either 90 or 120 mg at the discretion of attending physician. Tumour responses were routinely assessed every 2–3 months using CT or MRI and graded according to the Response Evaluation Criteria in Solid Tumours (RECIST) version 1.1 [13], and additional imaging studies were performed, as required, according to the condition of individual patients.

### Preparation of [^68^Ga]Ga-DOTA-TOC

[^68^Ga]Ga-DOTA-TOC was manually synthesized using a 1850 MBq ^68^Ge/^68^Ga generator (iThemba LABS, South Africa) and GMP grade DOTA-TOC (ABX, Germany). For the radioisotope labelling, purified 1126 ± 90 MBq of ^68^Ga^3+^ [14, 15] and 45 μg of DOTA-TOC, which was dissolved in a 1.0 M sodium acetate buffer, were added to the eluted ^68^Ga solution, and they were heated at 95 °C for 10 min. The reaction mixture was purified with a Sep-Pak Light C18 cartridge, and [^68^Ga]Ga-DOTA-TOC was collected in a sterile vial after passing through a 0.22-μm sterile filter [16].

The radiochemical yield (non-decay corrected) and final activity of the [^68^Ga]Ga-DOTA-TOC were 45.7 ± 8.8% and 397.7 ± 115.2 MBq, respectively. The synthesized [^68^Ga]Ga-DOTA-TOC was satisfactory according to European Pharmacopeia quality control criteria. The radiochemical purity of the [^68^Ga]Ga-DOTA-TOC was 98.2 ± 1.0%, and the proportions of radiochemical impurities, such as ^68^Ga in colloidal form and ^68^Ga^3+^ ion, were 0.6 ± 1.0% and 1.7 ± 1.1%, respectively.

### [^68^Ga]Ga-DOTA-TOC PET/CT image acquisition

For [^68^Ga]Ga-DOTA-TOC PET/CT imaging, 171.7 ± 26.8 MBq (4.6 ± 0.7 mCi) of [^68^Ga]Ga-DOTA-TOC was administered intravenously. After 61 ± 5 min, PET/CT imaging was performed using PET/CT scanners (GE Discovery PET/CT 690, 690 Elite, or 710; GE Medical Systems, Milwaukee, WI, USA) from the vertex to the upper thigh. CT imaging was initially done for the determination of the attenuation map and lesion localization (120 kVp, auto mA, 0.5 rotation time, 3.75-mm slice thickness). Following this, PET imaging was done in three-dimensional mode (3-min emission per bed position, 500-mm field of view). Images were reconstructed via three-dimensional iterative reconstruction using the VPFX-S algorithm (4 iterations, 18 subsets, 4.0-mm field width at half maximum, 192 × 192 matrix). Parametric PET images showing standardized uptake values (SUVs) based on patient body weight were generated.

### Analysis of [^68^Ga]Ga-DOTA-TOC PET/CT variables

[^68^Ga]Ga-DOTA-TOC PET/CT images were analysed visually and semi-quantitatively. For the visual variable, the Krenning score was applied [17]. The 5-point Krenning score scale is the most commonly used system by comparing liver and spleen uptake in NETs. To assess semi-quantitative variables, the multi-foci segmentation method was used (Syngo.Via; Siemens Healthcare, Knoxville, TN, USA) [18]. First, we drew a 10-cm^3^-sized spherical volume-of-interest (VOI) in the right lobe of the normal liver parenchyma. Then, the automatic segmentation method showed all lesions higher than the cutoff value (1.5 multiplied by mean SUV of the liver VOI plus 2 standard deviations). Among them, we manually removed physiologic high-uptake lesions, such as those of the spleen, liver, and adrenal gland. The maximum SUV (SUVmax), whole tumour volume (WTV), and tissue receptor expression (TRE, WTV multiplied by SUVmean) were automatically drawn by the software. When all lesions showed equal or lower than the cutoff SUV, WTV and TRE were defined as 0.0. The tumour-to-liver ratio (TLR) was calculated as SUVmax divided by SUVmean of the liver VOI.

### Statistical analysis

The primary endpoint was progression-free survival (PFS). PFS was defined as the time between the start date of lanreotide therapy and the date of disease progression according to the RECIST version 1.1 or any cause of death, whichever occurred first. Because the number of events for overall survival (OS) was too small to have enough statistical power, OS analysis was not performed in the present study.

Results are shown as frequencies with percentages for categorical data and means ± standard deviations or medians with ranges for continuous data. [^68^Ga]Ga-DOTA-TOC PET/CT variables were compared between patients with no previous treatment and those who had undergone previous treatment patients using the chi-square test (for categorical data) and the Mann-Whitney *U* test (for continuous data). All of the clinicopathological and [^68^Ga]Ga-DOTA-TOC PET/CT variables were dichotomized according to the median values or optimal cutoff values obtained by receiver operating characteristic (ROC) curve analysis. Survival outcomes were estimated using Kaplan-Meier analysis and compared using the log-rank test. Univariate and multivariate analyses of the prognostic variables for PFS were performed using Cox regression analysis and presented with hazard ratios (HRs) and 95% confidence intervals (CIs). A *p* value less than 0.05 was considered significant. All of the statistical analyses were performed using PASW Statistics for Windows, version 18 (SPSS Inc., Chicago, IL, USA).

## Results

### Patients

The patients’ baseline characteristics are summarized in Table 1. The mean age was 55.1 ± 15.5 year (range 16.0–81.0), and 38.7% of patients were male (*n* = 12). All patients had ECOG performance statuses of 0 (25.8%) or 1 (74.2%). The most common primary tumour sites were the pancreas (*n* = 17, 54.8%), stomach (*n* = 5, 16.1%), and rectum (*n* = 4, 12.9%). All tumours were histopathologically confirmed well-differentiated NETs. Most of the tumours were WHO grade 1 (*n* = 8, 25.8%) or 2 (*n* = 20, 64.5%) NETs, with a median Ki-67 index of 7.5% (range 0.5–25.0). Metastatic sites were the lymph nodes (*n* = 26, 83.9%), liver (*n* = 18, 58.1%), bone (*n* = 14, 54.2%), peritoneum (*n* = 5, 16.1%), lung (*n* = 4, 12.9%), duodenum (*n* = 2, 6.5%), spinal cord (*n* = 2, 6.5%), and pancreas (*n* = 1, 3.2%). Five patients received 90 mg of lanreotide, and the remaining 26 patients received 120 mg of lanreotide. At the time of analysis, 20 patients (64.5%) had experienced disease progression on lanreotide and 7 (22.5%) patients had died. The median PFS interval with lanreotide was 14.4 months (range 1.3–34.9).

**Table 1:** Patient profiles

Characteristic	Value
Total number of patients	31
Age (year)	55.1 ± 15.5 (16.0–81.0)
Gender (M:F)	12:19
ECOG performance status	
0	8 (25.8%)
1	23 (74.2%)
Primary tumour site	
Pancreas	17 (54.8%)
Stomach	5 (16.1%)
Small bowel	2 (6.5%)
Rectum	4 (12.9%)
Unknown	3 (9.7%)
Differentiation of tumour	
Well-differentiated	31 (100.0%)
NET WHO grade	
1	8 (25.8%)
2	20 (64.5%)
3	2 (6.5%)
Unknown	1 (3.2%)
Median Ki-67 index (%)	7.5 (0.5–25.0)
Existence of carcinoid symptoms	8 (25.8%)
Previous treatment	
Surgery	14 (45.2%)
Chemotherapy	4 (12.9%)
Hepatic tumour volume assessed by CT or MRI	
0–25%	15 (48.4%)
> 25%	10 (32.2%)
Unknown	6 (19.4%)
Best response after lanreotide therapy	
Partial response	1 (3.2%)
Stable disease	28 (90.3%)
Progressive disease	2 (6.5%)
Progression of disease after lanreotide therapy	20 (64.5%)
Median PFS after lanreotide therapy (months)	14.4 (1.3–34.9)

*ECOG* Eastern Cooperative Oncology Group, *NET* neuroendocrine tumour, *WHO* World Health Organization, *CT* computed tomography, *MRI* magnetic resonance imaging, *PFS* progression-free survival

### [^68^Ga]Ga-DOTA-TOC PET/CT variables

The tumour Krenning scores were 2 in 3 patients (9.7%), 3 in 6 patients (19.4%), and 4 in 22 patients (71.0%). The median TLR was 8.1 (range 1.0–36.9), and the median SUVmax was 42.9 (range 5.9–180.1). The median WTV was 58.9 cm^3^ (range 0.0–617.7), and the median TRE was 778.5 (range 0.0–9694.0). The mean liver SUVmean was 5.5 ± 1.7 (range 2.8–9.3). No significant difference was found between patients with no previous treatment and those who had undergone previous treatment (Table 2). In ROC curve analysis, TLR had the highest AUC (0.659) and Youden index J (0.4682) among all of the PET/CT variables, followed by SUVmax (AUC 0.632, Youden index J 0.4273) (Supplementary Tables 1-3).

**Table 2:** Summary and comparison of [^68^Ga]Ga-DOTA-TOC PET/CT results

Variable	Whole patients (*n* = 31)	No previous treatment (*n* = 15)	Previous treatment (*n* = 16)	*p* value
Krenning score (2/3/4)	3/6/22	1/4/10	2/2/12	0.562
Median tumour-to-liver ratio (TLR)	8.1 (1.0–36.9)	6.5 (1.7–36.9)	10.9 (1.0–36.1)	0.299
Median maximum standardized uptake value (SUVmax)	42.9 (5.9–180.1)	50.7 (6.8–180.1)	63.0 (5.9–143.1)	0.446
Median whole tumour volume (WTV, cm^3^)	58.9 (0.0–617.7)	71.2 (0.0–617.7)	26.1 (0.0–219.5)	0.216
Median total receptor expression (TRE)	778.5 (0.0–9694.0)	1186.1 (0.0–9694.0)	687.2 (0.0–5007.3)	0.188
Mean liver mean SUV (SUVmean)	5.5 ± 1.7 (2.8–9.3)	5.4 ± 1.7 (2.8–9.3)	5.6 ± 1.7 (2.8–8.6)	0.626

*PET* positron emission tomography, *CT* computed tomography

### Prognostic factor analyses

In the univariate analysis, ECOG performance status (1 vs 0; HR = 3.749 [95% CI 1.084–12.965], *p* = 0.037) was the only clinicopathological variable that was significantly associated with PFS (Table 3). Among the [^68^Ga]Ga-DOTA-TOC PET/CT variables, TLR (< 8.1 vs. ≥ 8.1; HR = 3.329 [95% CI 1.294–8.561], *p* = 0.013), SUVmax (< 42.94 vs. ≥ 42.94); HR = 2.656 [95% CI 1.059–6.663], *p* = 0.037), and WTV (≥ 58.9 cm^3^ vs. < 58.9 cm^3^; HR = 2.721 [95% CI 1.100–6.732], *p* = 0.030) were significantly associated with PFS in the univariate analyses (Table 4).

**Table 3:** Univariate analysis of clinicopathological variables for PFS after lanreotide therapy

Variable	Hazard ratio (95% CI)	*p* value
EGOG performance status (1 vs. 0)	3.749 (1.084–12.965)	0.037*
Primary tumour site (pancreas vs. other)	0.864 (0.0.356–2.097)	0.746
NET WHO grade (2 and 3 vs. 1)	1.833 (0.611–5.499)	0.279
Presence of carcinoid symptoms (yes vs. no)	0.616 (0.203–1.868)	0.392
Previous surgery (yes vs. no)	0.720 (0.290–1.789)	0.480
Previous chemotherapy (yes vs. no)	0.947 (0.217–4.133)	0.942
Lanreotide dose (90 mg vs. 120 mg)	2.030 (0.464–8.888)	0.347
Hepatic tumour volume assessed by CT/MRI (> 25% vs. 0–25%)	0.466 (0.170–1.275)	0.137

*CI* confidence interval

*Statistically significant (*p* < 0.05)

**Table 4:** Univariate analysis of [^68^Ga]Ga-DOTA-TOC PET/CT variables for PFS after lanreotide therapy

Variable	Hazard ratio (95% CI)	*p* value
Krenning score (2 and 3 vs. 4 )	1.876 (0.713–4.932)	0.202
Tumour-to-liver ratio (TLR) (< 8.1 vs. ≥ 8.1)	3.329 (1.294–8.561)	0.013*
Maximum standardized uptake value (SUVmax) (< 42.94 vs. ≥ 42.94)	2.656 (1.059–6.663)	0.037*
Whole tumour volume (WTV) (≥ 58.9 cm^3^ vs. < 58.9 cm^3^)	2.721 (1.100–6.732)	0.030*
Total receptor expression (TRE) (≥ 778.5 vs. < 778.5)	1.837 (0.0.749–4.506)	0.184

*Statistically significant (*p* < 0.05)

**Fig. 2:**
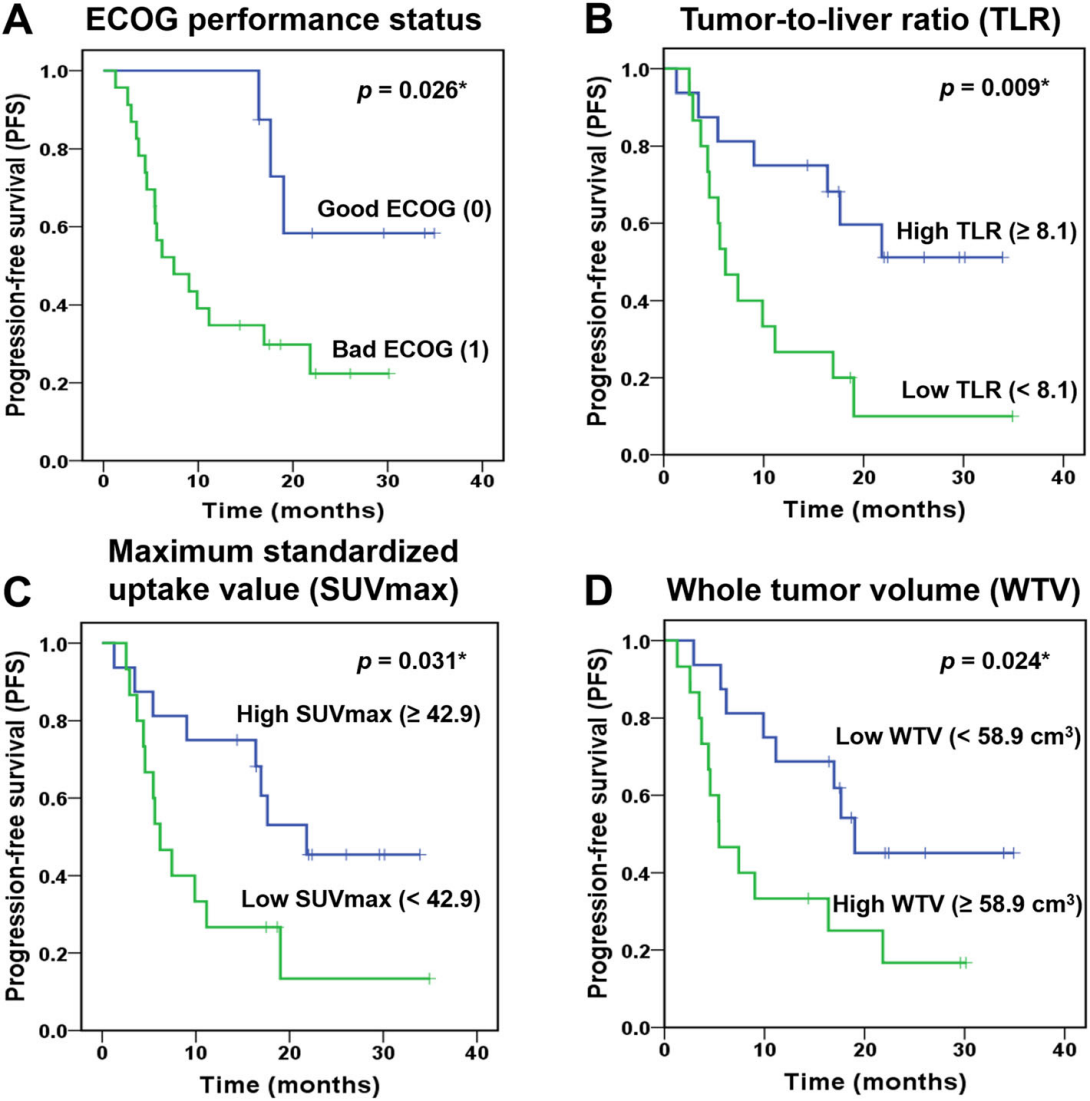
Kaplan-Meier analysis and log-rank test for PFS prediction after lanreotide therapy. **a** ECOG performance status (*p* = 0.026), **b** TLR (*p* = 0.009), **c** SUVmax (*p* = 0.031), and **d** WTV (*p* = 0.024) demonstrated significant results

**Fig. 3:**
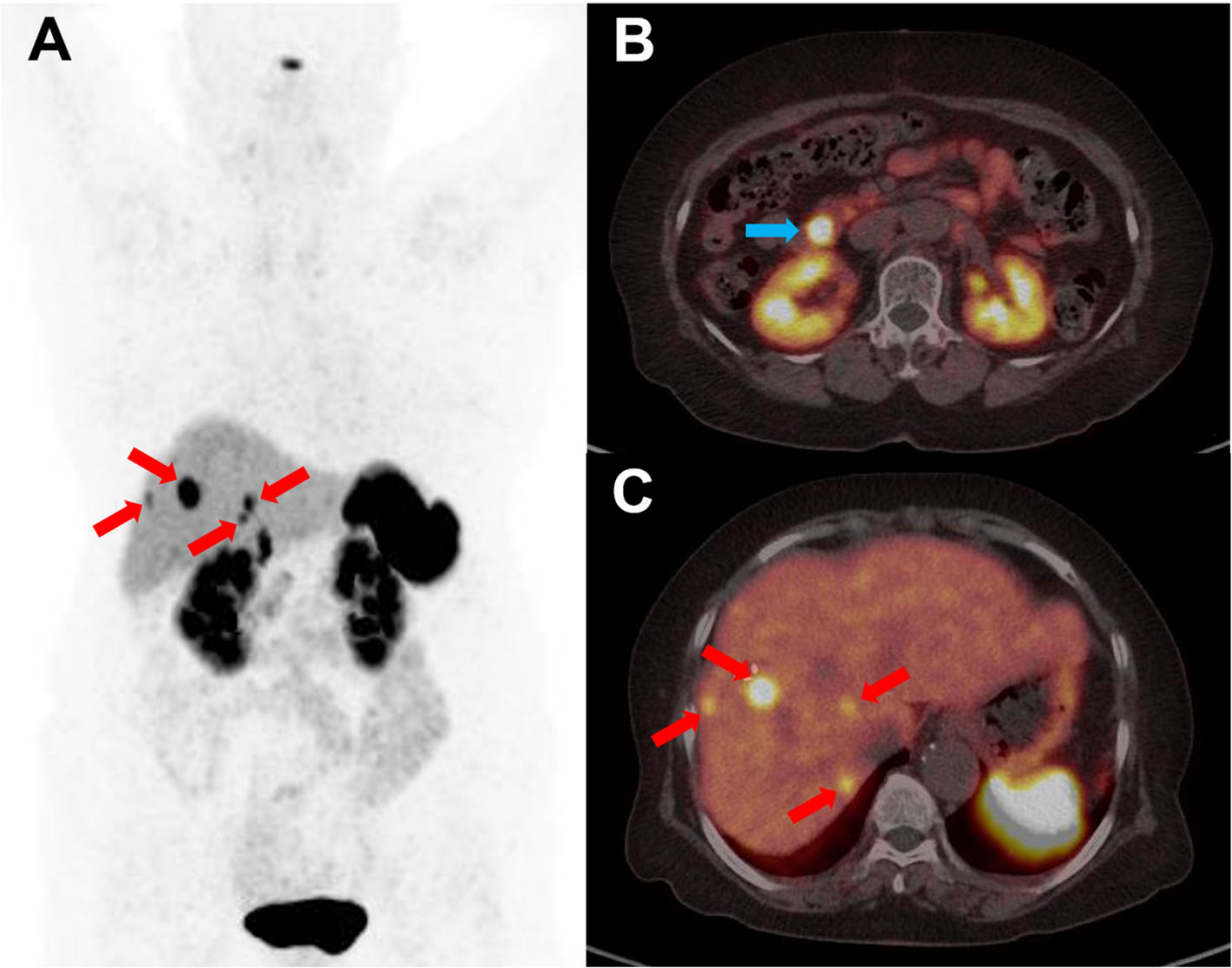
Representative case of a 77-year-old male with a primary duodenal NET grade 2. Maximal intensity projection (MIP, **a**) and trans-axial (**b**, **c**) [^68^Ga]Ga-DOTA-TOC PET/CT images show a primary NET at the duodenal second portion (blue arrow) and multiple small hepatic metastases (red arrows) with intense uptake. He had a good ECOG performance status (0), high TLR (11.8), high SUVmax (53.7), and low WTV (8.5), predicting a good prognosis. After lanreotide therapy, no progression was found until 34 months of follow-up

**Fig. 4:**
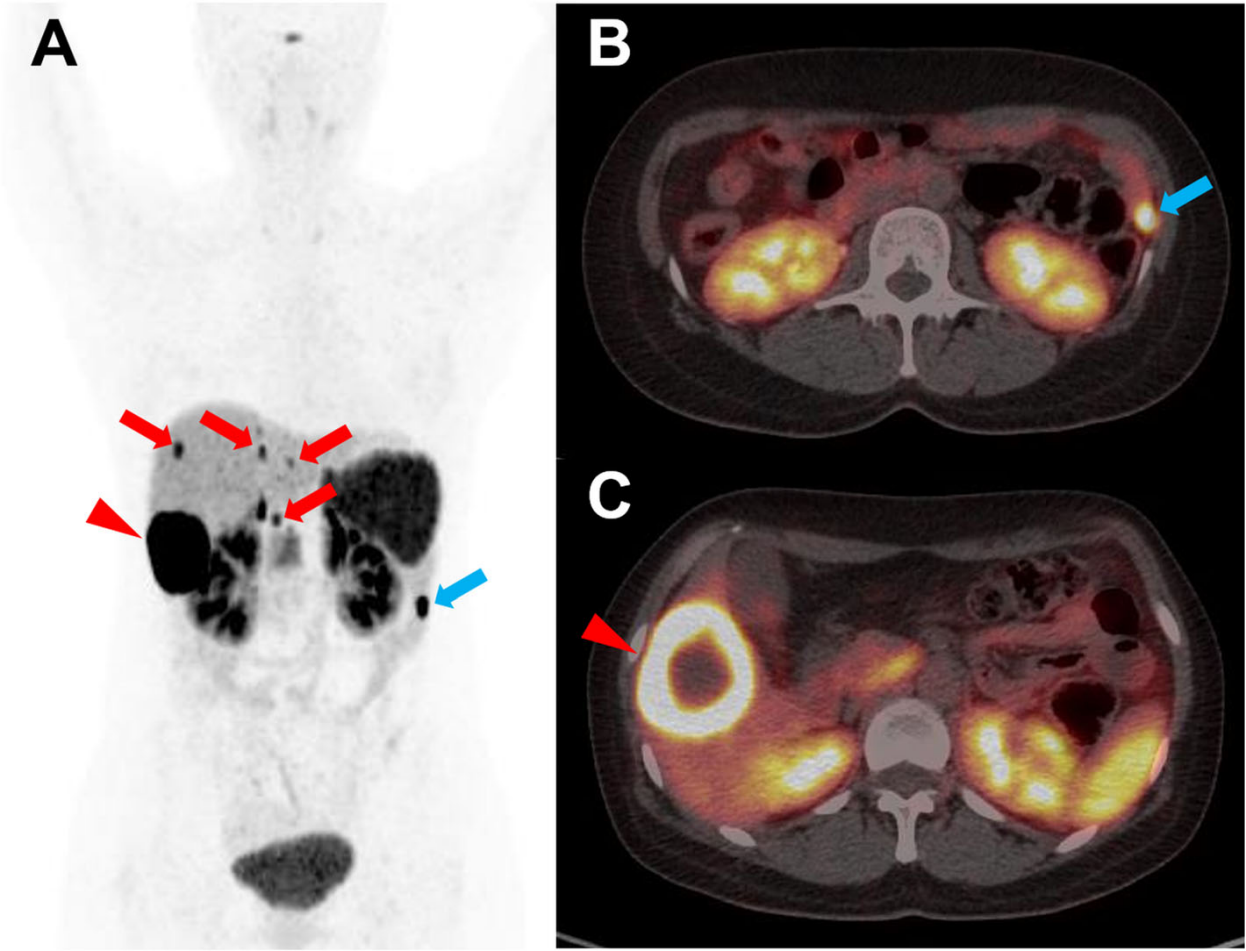
Representative case of a 37-year-old male with a primary jejunal NET grade 2. MIP (**a**) and trans-axial (**b**, **c**) [^68^Ga]Ga-DOTA-TOC PET/CT images reveal primary NET at the jejunal loop (blue arrow) and multiple small (red arrows) and large (red arrowhead) hepatic metastases with intense uptake. He had high TLR (17.7) and high SUVmax (77.5), predicting a good prognosis. However, he had a poor ECOG performance status (1) and high WTV (112.5), predicting poor prognosis. After lanreotide therapy, no progression was found until 30 months of follow-up

**Fig. 5:**
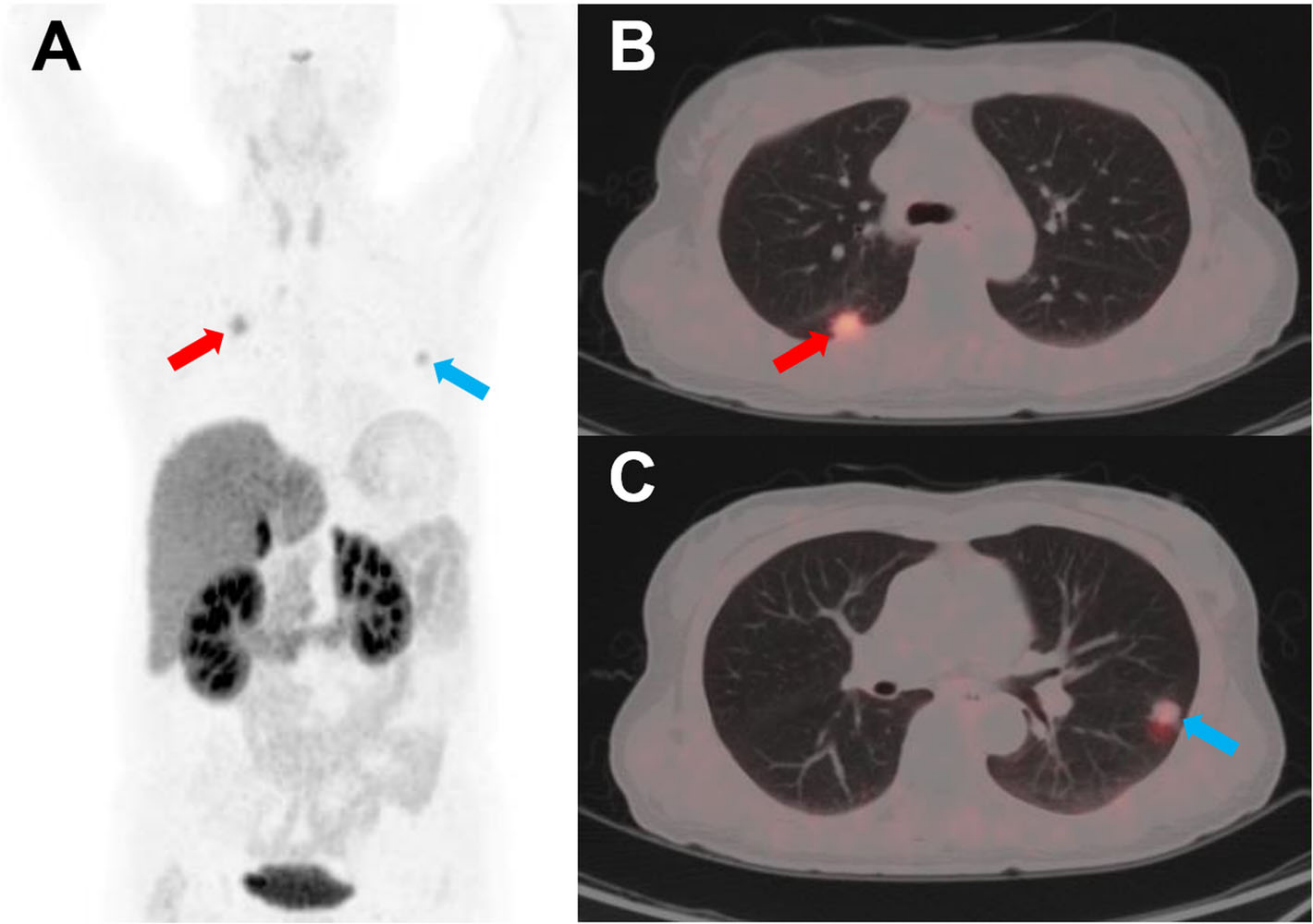
Representative case of a 55-year-old male with a previous primary pancreatic NET grade 2. The patient underwent surgery for the primary pancreatic NET 8 years prior, and multiple lung nodules were found in a follow-up chest CT image. MIP (**a**) and trans-axial (**b**, **c**) [^68^Ga]Ga-DOTA-TOC PET/CT images demonstrate small metastases with moderate uptake (red and blue arrows) in both lungs. His low TLR (0.98) and low SUVmax (5.85) predicted a poor prognosis; however, his good ECOG performance status (0) and low WTV (0.0) predicted a good prognosis. After lanreotide therapy, disease progression (metastatic nodule) was found in the right lower pulmonary lobe (red arrow; long-diameter increased from 1.2 to 1.7 cm) after 3 months of follow-up

In the multivariate analysis, TLR remained significantly associated with PFS (HR = 3.182 [95% CI 1.189–8.514], *p* = 0.021) when the impact of ECOG performance status was adjusted, while SUVmax and WTV were not statistically significant (Table 5). The mean PFS intervals according to the TLR (< 8.1 vs. ≥ 8.1) were 10.8 months (95% CI 5.8–15.8) and 23.0 months (95% CI 16.8–29.3), respectively (Fig. 2). Representative images are shown in Figs. 3, 4, and 5.

**Table 5:** Multivariate analysis of [^68^Ga]Ga-DOTA-TOC PET/CT variables with ECOG performance

Variable	Multivariate analysis I		Multivariate analysis II		Multivariate analysis III	
	Hazard ratio (95% CI)	*p* value	Hazard ratio (95% CI)	*p* value	Hazard ratio (95% CI)	*p* value
EGOG performance status (1 vs. 0)	3.518 (0.988–12.525)	0.052	3.528 (1.000–12.454)	0.050	3.188 (0.908–11.191)	0.070
Tumour-to-liver ratio (TLR) (< 8.1 vs. ≥ 8.1)	3.182 (1.189–8.514)	0.021*	NA	NA	NA	NA
SUVmax (< 42.9 vs. ≥ 42.9)	NA	NA	2.493 (0.960–6.475)	0.061	NA	NA
Whole tumour volume (WTV) (≥ 58.9 cm^3^ vs. < 58.9 cm^3^)	NA	NA	NA	NA	2.294 (0.917–5.743)	0.076

*NA* not assessed

*Statistically significant (*p* < 0.05)

## Discussion

The TLR determined by [^68^Ga]Ga-DOTA-TOC PET/CT may be a useful prognostic factor associated with the efficacy of lanreotide for treating unresectable or metastatic WD GEP-NETs, with low TLRs being associated with shorter PFS with lanreotide.

1The prognostic value of SSTR imaging among patients receiving somatostatin analogues is currently not well understood. A recent meta-analysis assessing the prognostic value of SSTR PET/CT revealed significance in terms of PFS and OS [10]. However, many previous studies have used heterogeneous radiotracers [19, 20], and no study has investigated a single radiotracer of ^68^Ga-DOTA-TOC. Additionally, treatment has been heterogeneous, including surgery, somatostatin analogues, peptide receptor radionuclide therapy (PRRT), and everolimus [21–23]. Only one study reported the prognostic value of a single somatostatin analogue therapy (octreotide) associated with a single [^68^Ga]Ga-DOTA-conjugated radiotracer ([^68^Ga]Ga-DOTA-TATE) [24].

Interestingly, the TLR determined by [^68^Ga]Ga-DOTA-TOC PET/CT was significantly associated with PFS in association with lanreotide in our study, whereas many previous studies have demonstrated SUVmax as a significant prognostic factor in association with SSTR PET/CT [21–24]. There has been some debate on the evaluation of [^18^F]FDG PET/CT tumour SUVmax, as tumour heterogeneity, image acquisition time, and body size may hamper the ability of SUVmax to accurately reflect tumour characteristics [25]. Additionally, a single SUVmax pixel may not represent the entire tumour, and a partial volume effect can be induced [26]. Furthermore, the [^68^Ga]Ga-DOTA-TOC PET/CT tumour SUVmax could also be affected by factors other than the degree of tumour SSTR expression, such as peptide mass [27], splenectomy status [28], and previous treatment including somatostatin analogue therapy [29, 30]. Therefore, in a previous study, the changes in tumour SUVmax normalized by the SUVmean of the spleen variable (delta tumour-to-spleen ratio) was suggested as a potentially useful factor to use instead of the delta tumour SUVmax for predicting the PRRT response with [^68^Ga]Ga-DOTA-TOC PET/CT [31]. Similarly, the [^68^Ga]Ga-DOTA-TOC TLR in our study might reduce the interscan variability by using normal liver uptake as an internal reference of tumour uptake. The normal liver uptake of [^68^Ga]Ga-DOTA-TOC is known to be less affected by other factors and less variable than spleen uptake [30, 32].

Volumetric variables (WTV and TRE) determined by [^68^Ga]Ga-DOTA-conjugated radiotracer PET/CT were assessed in a previous article, which revealed that the WTV determined by [^68^Ga]Ga-DOTA-TATE PET/CT is associated with prognosis [33]. However, a limitation of this study was the heterogeneous patient population in terms of treatment, including surgery, PRRT, liver-directed therapy, and systemic therapy. Therefore, the study findings may not accurately reflect the prognostic implications of WTV for specific treatment. To date, TRE has not been considered a prognostic factor, and our results support the previous findings. This may be due to the opposite impact on prognosis between SUVs and WTV in terms of SSTR PET/CT. As low SUVs and high WTVs predict a poor prognosis, SUVmean multiplied by WTV variable (TRE) may not have a relevant prognostic performance.

Our findings suggest that the low TLR determined by [^68^Ga]Ga-DOTA-TOC PET/CT may be a factor of worse prognosis of outcomes associated with lanreotide. This imaging feature may be associated with the clinical implications of treatment monitoring and therapeutic decision-making. More frequent follow-up imaging assessment may be needed for patients treated with lanreotide who have the low TLR at baseline [^68^Ga]Ga-DOTA-TOC PET/CT. Furthermore, the TLR may be used to stratify the therapeutic approaches for GEP-NET patients whose lanreotide treatment failed. Stemming from the success of phase III clinical trials (RADIANT-3 and 4 and NETTER-1) [34–36], everolimus and PRRT ([^177^Lu]Lu-DOTA-TATE) are currently the standard of care for lanreotide-progressed GEP-NET patients. As PRRT is also an SSTR-targeted therapy, the high TLR might be a good indicator favouring PRRT over everolimus. However, this should be further evaluated in future studies including patients treated with PRRT.

Some limitations exist in our study. First, the study was retrospective, and it included a small number of patients from a single center. We believe that well-known prognostic factors, such as hepatic tumour volume, demonstrated no significant results because of the small sample size. However, our results might suggest that the TLR, as determined by [^68^Ga]Ga-DOTA-TOC PET/CT, is a more powerful prognostic factor than the hepatic tumour volume, viewed from the other side. Second, some included patients had more advanced or aggressive disease than that which normally calls for first-line treatment with lanreotide; therefore, disease status or previous therapy may have affected the results. Third, we could not perform the analysis for the prognostic value of ^68^Ga-DOTA-TOC PET/CT in terms of OS because of small number of events to input into the OS calculation. Future prospective multicentre studies with larger sample sizes are needed.

## Conclusions

In conclusion, the TLR yielded by [^68^Ga]Ga-DOTA-TOC PET/CT has prognostic value for patients with unresectable or metastatic WD GEP-NETs who received lanreotide therapy. Low TLRs are associated with worse prognoses. Further investigations of the implications of the TLR in the management of GEP-NETs are needed in terms of predictive markers or indicators for therapeutic decisions.

## Supplementary information

**Additional file 1: Supplemenatry Table 1.** ROC curve analysis of [^68^Ga]Ga-DOTA-TOC PET/CT variables for PFS after lanreotide therapy. **Supplementary Table 2.** Univariate analysis of [^68^Ga]Ga-DOTA-TOC PET/CT variables for PFS after lanreotide therapy according to optimal cutoff of ROC curve analysis. **Supplementary Table 3.** Multivariate analysis of [^68^Ga]Ga-DOTA-TOC PET/CT variables with ECOG performance according to optimal cutoff of ROC curve analysis.

## Data Availability

The datasets used and/or analysed during the current study are available from the corresponding author on reasonable request.

## Abbreviations

NET: Neuroendocrine tumour
GEP: Gastroenteropancreatic
SSTR: Somatostatin receptor
WD: Well-differentiated
PET: Positron emission tomography
CT: Computed tomography
ECOG: Eastern Cooperative Oncology Group
WHO: World Health Organization
MRI: Magnetic resonance imaging
RECIST: Response evaluation criteria in solid tumours
VOI: Volume-of-interest
SUV: Standardized uptake value
WTV: Whole tumour volume
TRE: Tissue receptor expression
TLR: Tumour-to-liver ratio
PFS: Progression-free survival
OS: Overall survival
HR: Hazard ratio
CI: Confidence interval
PRRT: Peptide receptor radionuclide therapy
