# Supplementary material for: Tumour-to-liver ratio determined by [68Ga]Ga-DOTA-TOC PET/CT as a prognostic factor of lanreotide efficacy for patients with well-differentiated gastroenteropancreatic-neuroendocrine tumours
Source: EJNMMI Res. 2020 Jun 15;10:63. doi: 10.1186/s13550-020-00651-z (PMC7295884; doi:10.1186/s13550-020-00651-z)
Supplement: Supplementary file 1 — Additional file 1: Supplemenatry Table 1. ROC curve analysis of [68Ga]Ga-DOTA-TOC PET/CT variables for PFS after lanreotide therapy. Supplementary Table 2. Univariate analysis of [68Ga]Ga-DOTA-TOC PET/CT variables for PFS after lanreotide therapy according to optimal cutoff of ROC curve analysis. Supplementary Table 3. Multivariate analysis of [68Ga]Ga-DOTA-TOC PET/CT variables with ECOG performance according to optimal cutoff of ROC curve analysis. [file 13550_2020_651_MOESM1_ESM.docx]

**SUPPLEMENTARY TABLES**

**Supplemenatry Table 1.** ROC curve analysis of [^68^Ga]Ga-DOTA-TOC PET/CT variables for PFS after lanreotide therapy

| Variable | AUC (95% CI) | *p* value | Youden index J | Cutoff | Sensitivity (%) | Specificity (%) |
| --- | --- | --- | --- | --- | --- | --- |
| Krenning sore | 0.598 (0.407 - 0.769) | 0.2146 | 0.1682 | 3 | 35.00 | 81.82 |
| TLR | 0.659 (0.468 - 0.819) | 0.1183 | 0.4682 | 7.77 | 65.00 | 81.82 |
| SUVmax | 0.632 (0.440 - 0.769) | 0.2286 | 0.4273 | 52.38 | 70.00 | 72.73 |
| WTV | 0.586 (0.396 - 0.759) | 0.4147 | 0.3773 | 32.84 | 65.00 | 72.73 |
| WTV-to-liver ratio | 0.595 (0.405 - 0.767) | 0.3674 | 0.3773 | 5.43 | 65.00 | 72.73 |
| TRE | 0.568 (0.379 - 0.744) | 0.5179 | 0.3273 | 778.47 | 60.00 | 72.73 |
| TRE-liver ratio | 0.577 (0.388 - 0.752) | 0.4693 | 0.3773 | 128.67 | 65.00 | 72.73 |

ROC = receiver operating characteristic; PET = positron emission tomography; CT = computed tomography; PFS = progression-free survival; AUC = area under the curve; TLR = tumour-to-liver ratio; SUVmax = maximum standardized uptake value; WTV = whole tumour volume; TRE = total receptor expression

**Supplementary Table 2.** Univariate analysis of [^68^Ga]Ga-DOTA-TOC PET/CT variables for PFS

after lanreotide therapy according to optimal cutoff of ROC curve analysis

| Variable | Hazard ratio (95% CI) | *p* value |
| --- | --- | --- |
| Krenning score (2 & 3 vs. 4 ) | 1.876 (0.713–4.932) | 0.202 |
| TLR  (≤ 7.77 vs. > 7.77) | 3.329 (1.294–8.561) | 0.013* |
| SUVmax  (≤ 52.38 vs. > 52.38) | 3.504 (1.303–9.425) | 0.013* |
| WTV (> 32.84 cm^3^ vs. ≤ 32.84 cm^3^) or  WTV-to-liver ratio (> 5.43 cm^3^ vs. ≤ 5.43 cm^3^) or  TRE-to-liver ratio (> 128.67 vs. ≤ 128.67) | 2.774 (1.099–7.002) | 0.030* |
| TRE (> 778.47 vs. ≤ 778.47) | 1.837 (0.0.749–4.506) | 0.184 |

* Statistically significant (*p* < 0.05)

**Supplementary Table 3.** Multivariate analysis of [^68^Ga]Ga-DOTA-TOC PET/CT variables with ECOG performance according to

optimal cutoff of ROC curve analysis

| Variable |  | Multivariate analysis I | |  | Multivariate analysis II | |  | Multivariate analysis III | | |
| --- | --- | --- | --- | --- | --- | --- | --- | --- | --- | --- |
|  |  | Hazard ratio (95% CI) | *p* value |  | Hazard ratio (95% CI) | *p* value |  | Hazard ratio (95% CI) | *p* value |  |
| EGOG performance status  (1 vs. 0) |  | 3.518 (0.988 - 12.525) | 0.052 |  | 3.265 (0.913 - 11.681) | 0.069 |  | 3.520 (1.015 - 12.206) | 0.047* |  |
| TLR (< 8.1 vs. ≥ 8.1) |  | 3.182 (1.189 - 8.514) | 0.021* |  | NA | NA |  | NA | NA |  |
| SUVmax (< 42.9 vs. ≥ 42.9) |  | NA | NA |  | 3.179 (1.124 - 8.992) | 0.029* |  | NA | NA |  |
| WTV  (> 32.84 cm3 vs. ≤ 32.84 cm3) or WTV-to-liver ratio  (> 5.43 cm3 vs. ≤ 5.43 cm3) or TRE-to-liver ratio (> 128.67 vs. ≤ 128.67) |  | NA | NA |  | NA | NA |  | 2.611 (1.030 - 6.621) | 0.043* |  |

NA = not assessed

* Statistically significant (*p* < 0.05)
